# Rapamycin Improved Retinal Function and Morphology in a Mouse Model of Retinal Degeneration

**DOI:** 10.3389/fnins.2022.846584

**Published:** 2022-02-28

**Authors:** Meng Zhao, Houting Lv, Na Yang, Guang-Hua Peng

**Affiliations:** ^1^Laboratory of Visual Cell Differentiation and Regulation, Basic Medical College, Zhengzhou University, Zhengzhou, China; ^2^Department of Pathophysiology, Basic Medical College, Zhengzhou University, Zhengzhou, China

**Keywords:** retinal degeneration, MTOR, rapamycin, photoreceptor, autophagy

## Abstract

The retina is an important visual organ, which is responsible for receiving light signals and transmitting them to the optic nerve center step by step. The retina contains a variety of cells, among which photoreceptor cells receive light signals and convert them into nerve signals, and are mainly responsible for light and dark vision. Retinal degeneration is mainly the degeneration of photoreceptor cells, and retinitis pigmentosa (RP) is characterized by rod degeneration followed by cone degeneration. So far, there is still a lack of effective drugs to treat RP. Here, we established a stable RP model by tail vein injection of methyl methanesulfonate to study the mechanism of retinal photoreceptor degeneration. Mechanistic target of rapamycin (mTOR) is located in the central pathway of growth and energy metabolism and changes in a variety of diseases in response to pathological changes. We found that the mTOR was activated in this model. Therefore, the inhibitor of mTOR, rapamycin was used to suppress the expression of mTOR and interfere with photoreceptor degeneration. Electroretinogram assay showed that the function of mice retina was improved. Hematoxylin and eosin staining results displayed that retinal photoreceptor thickness and morphology were improved. Also, the autophagy in rapamycin group was activated, which revealed that rapamycin may protect the retinal photoreceptor by inhibiting mTOR and then activating autophagy.

## Introduction

Retina is an important component in the process of vision formation. The retina is responsible for converting the incoming optical signals into electrical signals, which are finally transmitted to the central nervous system (Masland, 2003). The retina is mainly composed of pigment epithelial cells, photoreceptor cells, horizontal cells, bipolar cells, amacrine cells, and ganglion cells (Hoon et al., 2014). Photoreceptor cells are responsible for converting light signals into nerve signals. It includes cone cells responsible for bright vision and rod cells responsible for dark vision. Retinitis pigmentosa (RP) is a hereditary degeneration of retinal photoreceptors, in which the retinal rod cells die first, followed by the retinal cones (Hartong et al., 2006). The symptoms of RP are disappearance of night vision, followed by disappearance of central vision, up to complete blindness (Koch et al., 2015). The incidence rate of RP worldwide is about 1/4,000, which seriously affects the quality of life of patients. Although more than 100 genes related to RP have been discovered, there is no effective treatment for RP yet (Zhao et al., 2020).

In the study of the progress and treatment of RP, in addition to gene mutant mice models such as rd1 and rd10 (Chang et al., 2002), there are some convenient and rapid drug-induced RP models such as *N*-Methyl-*N*-nitrosourea (MNU) and sodium iodate (NaIO3) induced photoreceptor degeneration mice (Machalinska et al., 2010; Tao et al., 2015). It was found that SN2 DNA alkylating agent methyl methanesulfonate (MMS) can induce retinal photoreceptor cell apoptosis and severe retinal degeneration in chicken embryos (Karran et al., 1977). In mice, MMS can specifically damage mouse retinal photoreceptor cells. The main mechanism of this is the initiation of the base excision repair (BER) pathway through 3-methyladenine DNA glycosylase (AAG) (Meira et al., 2009). Compared to female mice, male mice were more sensitive to MMS induced photoreceptor degeneration (Allocca et al., 2019). According to above researches and the previous experiments conducted in our laboratory, we established a stable mouse model of retinal photoreceptor degeneration induced by single tail vein injection of MMS.

Mechanistic target of rapamycin (mTOR) pathway plays an important role in regulating cell cycle, protein synthesis, energy metabolism and autophagy under normal conditions (Liu and Sabatini, 2020). mTOR belongs to serine threonine kinase of phosphoinositide-3-kinase (PI3K) related kinase family and is the central component of mTOR signaling pathway (Zhang et al., 2020). mTOR can form two main mTOR complexes to perform biological functions: mTORC1 mainly regulates cell metabolism in response to nutrient availability, growth factor, energy and stress, while mTORC2 modulates actin cytoskeletal organization and cell polarization in response to growth factors (Yang et al., 2013).

Recent studies have found that the abnormal expression of mTOR is related to a variety of ophthalmic diseases, and the inhibition of mTOR with rapamycin has produced some effects (Yao and van Wijngaarden, 2020). For example, Hayashi et al. (2015) concluded that rapamycin may protect retinal ganglion cells from *N*-Methyl-D-aspartic Acid (NMDA) induced injury by activating the ERK pathway of Müller cells. Zhao et al. (2011) found that inhibition of mTOR could improve retinal photoreceptor cell death caused by retinal pigment epithelial (RPE) pressure. Li et al. (2014) showed that rapamycin could protect retinal photoreceptor cells from visible light stimulation by inhibiting endoplasmic reticulum stress. Fan et al. (2016a) demonstrated that inhibition of mTOR with rapamycin reduced photoreceptor cell death caused by serum deficiency, which may be related to the changes in intracellular reactive oxygen species level, oxidative stress, mitochondrial function and G2/M cell cycle arrest. Fan et al. (2016b) also found that rapamycin could improve the effect of glucose deprivation on photoreceptor cells by restoring mitochondrial function. Yang et al. (2021) discovered that the expression of mTOR increased at postnatal day 10 and the inhibition of mTOR with rapamycin slowed down the degeneration process of photoreceptor cells and improved retinal function in rd1 mice.

In this study, mTOR was activated in MMS induced photoreceptor degeneration model. Through intraperitoneal injection of mTOR inhibitor rapamycin, we showed that rapamycin significantly improved mouse retinal function, improved mouse behavioral activities and delayed the degeneration process of photoreceptor cells.

## Materials and Methods

### Animals and Treatment

The used animal was C57BL/6J male mice which were 7–8 weeks old in this study. All animals were maintained under standard laboratory conditions (room temperature 17–25°C, humidity 40–60%, 12 h dark/light cycle) with free food and water. All experiments with animals were approved by the Animal Research Ethics Committee of Zhengzhou University in accordance with administrative regulations for animal experimental in China. Project identification code was 2020-ky-67, and the date of approval was July 10, 2020. Much efforts were made to reduce the number of mice and their suffering. The initial density of MMS (M4882E961, ACMEC, China) is 1.3 g/ml. The injection method of MMS was single tail vein injection of 60 mg/kg, 5 μL/g body weight. The experiment of retinal degeneration induced by MMS was divided into two groups: Normal (*n* = 6) and MMS (1 day, *n* = 6; 2 day, *n* = 6; 3 day, *n* = 6; 4 day, *n* = 6; 5 day, *n* = 6; 7 day, *n* = 6; 10 day, *n* = 6). The injection method of rapamycin was intraperitoneal injection of 10 mg/kg, 10 μL/g body weight. Rapamycin was injected 1 day before MMS injection and every day until the fifth day after MMS injection. The experiment of rapamycin treatment was divided into four groups: Normal + PBS (*n* = 6), MMS + PBS (*n* = 6), Normal + Rapamycin (*n* = 6), and MMS + Rapamycin (*n* = 6).

### Electroretinogram Recording

All the mice were placed in the test room in advance, dark adapted for 12 h or more. Turn on the RETI-scan visual electrophysiological examination system (Roland Consult, Germany). The mice were placed in the isoflurane gas filled chamber to induce anesthesia. After the mice were anesthetized, they were fixed on the operating platform. The anesthesia mask was placed over their mouth and nose to maintain the deep anesthesia state. The breathing was stable, and the compound tropicamide eye drops were applied for mydriasis. Installation of electrodes: the grounding electrode was inserted into the subcutaneous tissue of mouse tail, two reference electrodes were inserted into the subcutaneous tissue of bilateral cheek, and two corneal ring electrodes were placed at the equator of eyeball, which could not compress eyeball. The mouse operating platform was pushed into the ganzfeld spherical stimulator to detect the five items of ERG proposed by the international society for clinical electrophysiology of vision (ISCEV).

### Histological Analysis

The fixed eyeball was washed with pure water, placed in a plate, and operated under a stereo-microscope (Olypus/SZ61). The cornea, lens and iris tissues were removed, and the optic cup was retained. After conventional gradient alcohol dehydration, xylene transparency and wax immersion (SAKURA/VIP-5-J r-JC2), the eyeball was embedded in paraffin (SAKURA/TEC 5 EM JC-2). Paraffin tissue was cut into 4 μm thick sections (YAMATO/RX-860). Every three tissue sections were pasted on one slide, and three slides were prepared for each eyeball. The slices were baked at 65°C for 60 min, dewaxed with xylene, hydrated with gradient alcohol, soaked in pure water for 5 min, stained with hematoxylin for 20 s, differentiated with hydrochloric acid, blue returned with ammonia, stained with eosin for 10 s, dehydrated with gradient alcohol, transparent with xylene and sealed with neutral resin. Under the microscope (Olympus/BX53), the retina with 300 μm of myopic nerve was photographed at 400× and the parts of retina were photographed at 200× with Photoshop 5.0 software. The distance between the upper end of the outer retinal nuclear layer and RPE was measured by Image J software.

### Open Field Test

The mice were placed in the normal light of the test room for 2 h in advance. Open the computer’s topscan version 3.0 software for program settings. The open field area was divided into peripheral area, central area and total area, and the activity time of mice was set as 600 s per mouse. Each subject was placed in the center area of the open field apparatus. Total distance traveled (mm), time duration in the center area and peripheral area (s), velocity in the open field (mm/s) and bounts in the center area were recorded automatically. After testing a mouse, disinfect the test area with 75% alcohol and dry it, and then carry out the next target test.

### Light/Dark Transition Test

The mice were placed in the normal light of the test room for 2 h in advance. Open the computer’s topscan version 3.0 software for program settings. The activity area was divided into Dark area and Light area, and the activity time of mice was set as 600 s per mouse. Each subject was placed in the Light chamber. Total distance traveled (mm), time duration in the Dark area and Light area (s), bounts in the Light area and proportion of time in black chamber and white chamber were recorded automatically. After testing a mouse, disinfect the test area with 75% alcohol and dry it, and then carry out the next target test.

### TUNEL Staining

Paraffin sections of retinal tissue were routinely dewaxed to water. Immunohistochemistry pen was used to circle around the retinal tissue. 20 μg/ml protease K without DNase (st532, Beyotime, China) was dripped. The cells were incubated at room temperature for 30 min and washed with PBS for 5 min × 3 times. Prepare the TUNEL detection solution (TDT enzyme: fluorescent labeling solution = 1:9), add 20 μL to each retina, put the slices into a wet box containing water, incubate them at 37°C for 60 min in dark, and wash them with PBS for 5 min × 3 times. Each tissue was incubated with 10 μl DAPI at room temperature for 3 min, washed with PBS for 5 min × 3 times, sealed with anti-fluorescence quenching solution, observed and took photos under fluorescence microscope.

### Western Blot

The retina protein was extracted using RIPA buffer (P0013B, Beyotime, China) with PMSF and phosphatase inhibitors. The concentration of extracted protein was measured with BCA protein concentration assay kit (P0009, Beyotime, China). According to the measured value, the samples were adjusted to the same protein concentration (0.8 μg/μL) and denatured. 15 μL of each sample containing equal amounts of protein (12 μg) was taken for electrophoresis and transferred. The PVDF membrane with Western blotting was blocked with blocking solution (5% skimmed milk powder prepared with TBST) for 2 h, and then western blotting was incubated with primary antibody mTOR, 1:1,000 (Abcam), p-mTOR, 1:1,000 (Abcam), Rhodopsin, 1:500 (Abcam), and GAPDH, 1:10,000 (Prteintech) overnight at 4°C. After overnight, the membrane was washed three times with TBST and then incubated with goat anti-mouse IgG secondary antibodies 1:10,000 (Proteintech) and goat anti-rabbit IgG secondary antibodies 1:10,000 (Proteintech). After washed three times with TBST, the PVDF membrane was added with the ECL droplet to make it uniformly distribute on the surface of the whole membrane and was put into the gel imaging system to take photos.

### Transmission Electron Microscope

Extracted the retina under the microscope, quickly cut the retina into 1 mm^3^ tissue and put it in the electron microscope fixing solution. Wash the tissue with 0.1M phosphate buffer PB (PH7.4) for three times, each time 15 min. The tissue was fixed with 1% OsO4 at room temperature and away from light for 2 h. The tissue was put in 30–50–70–80–95–100–100% ethanol for upward dehydration for 20 mins each time. Dehydrate with 100% acetone twice for 15 mins each time. Infiltrate the embedded tissue and overnight in a 37° oven. The embedded tissue was polymerized in a 60° oven for 48 h, and the resin block was used for slicing. Sliced the resin block and picked the piece with copper mesh. The sections were stained with citric acid and dried overnight. Took pictures with transmission electron microscope (HT7800/HT7700, Hitach) and saved the pictures.

### Statistical Analysis

Statistical analysis was performed using GraphPad Prism Version 7.0 software (Graph Pad software, San Diego, CA, United States). All measurement results are represented as Mean ± SD. One-way ANOVO was used for comparison of the effect of MMS at different time. Two-way ANOVO was used to evaluate the effect of rapamycin and MMS at the same time. *P* < 0.05 was considered statistically significant for all tests.

## Results

### The Outer Nucleus Layer Became Thinner Gradually After Tail Vein Injection of Methyl Methanesulfonate

**FIGURE 1:**
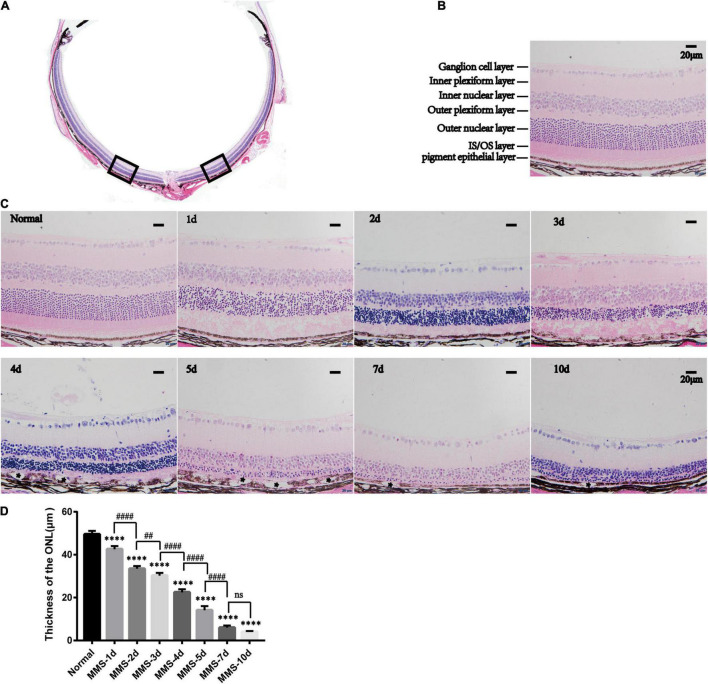
The retinal morphology changes with time after tail vein injection of methyl methanesulfonate (MMS). **(A)** The Panorama of retina. **(B)** The main layers of retina. **(C)** The normal retina without MMS administration and the retina 1, 2, 3, 4, 5, and 7 days after MMS administration. The asterisk represents the posterior vacuole structure in RPE cells. **(D)** The histogram of the thickness of outer nucleus layer (ONL). One way ANOVA multiple comparisons was analyzed, ^∗∗∗∗^*P* < 0.0001 for differences compared with controls; ^##^*P* < 0.01, ^####^*P* < 0.0001 for differences compared with previous time point group, *n* = 3.

We took mouse eyeballs at day 1, day 2, day 3, day 4, day 5, day 7, and day 10 for fixation, paraffin embedding, paraffin sectioning and hematoxylin and eosin (HE) staining to obtain the morphological changes of retinal photoreceptor cells at different time points after MMS injection (Figure 1). The hemispheric section of retina is displayed (Figure 1A), and the position about 200 μm on both sides of the optic nerve is used to calculate the thickness of the retinal outer nucleus layer (ONL) where photoreceptor cells are located. The retinal cells were labeled in 40 fold HE sections (Figure 1B). One way ANOVO analysis (*F* = 616.9, *P* < 0.0001) revealed that the thickness of the ONL layer decreased at day 1, day 2, day 3, day 4, day 5, day 7, and day 10 after MMS injection compared with normal retina (Figure 1D), and the ONL was the thinnest 10 days after MMS injection, *P* < 0.0001 (Figures 1C,D). In addition to the gradual thinning of the ONL of the retina, RPE cells showed posterior vacuole structure at 4, 5, 6, and 7 days after MMS injection (asterisk), which also suggested that pigment epithelial cells also showed stress response with the damage of photoreceptor cells (Figure 1C).

### The Retinal Function of Mice Deteriorated Rapidly After Tail Vein Injection of Methyl Methanesulfonate

**FIGURE 2:**
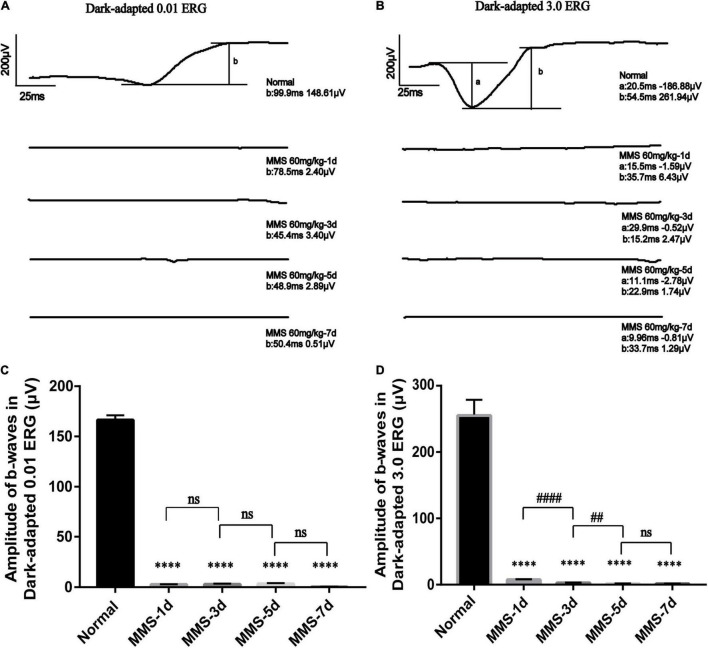
The retinal function deteriorates with time after tail vein injection of MMS. **(A)** The b-wave of Dark-adapted 0.01 electroretinogram (ERG) of normal retina without MMS treatment and retina after MMS administration (1, 3, 5, and 7 days). **(B)** The b-wave of Dark-adapted 3.0 ERG of normal retina without MMS treatment and retina after MMS administration (1, 3, 5, and 7 days). **(C)** The histogram of the amplitude of b-wave of Dark-adapted 0.01 ERG. One way ANOVA multiple comparisons was analyzed, ^∗∗∗∗^*P* < 0.0001 for differences compared with controls, *n* = 3. **(D)** The histogram of the amplitude of b-wave of Dark-adapted 3.0 ERG. One way ANOVA multiple comparisons was analyzed, ^∗∗∗∗^*P* < 0.0001 for differences compared with controls; ^####^*P* < 0.0001, ^##^*P* < 0.01 for differences compared with previous time point group, *n* = 3.

We performed a full-field (Ganzfeld) flash electroretinogram (ERG) test at day 1, day 3, day 5, and day 7 to record the changes of retinal photoreceptor function (Figure 2). The amplitude of the ERG wave can show the function of the whole visual conduction circuit from retinal photoreceptor cells to retinal ganglion cells (Robson et al., 2018). The b-wave of dark adapted 0.01 ERG (flash strength is 0.01 photopic cd^▪^s^▪^m^–2^ with a scotopic strength of 0.025 scotopic cd^▪^s^▪^m^–2^) is mainly produced by rod bipolar cells in the retina, which is the main index to detect the function of rod cells. The b-wave of dark-adapted 3.0 ERG (flash strength is 3.0 photopic cd^▪^s^▪^m^–2^ with a scotopic strength of 7.5 scotopic cd^▪^s^▪^m^–2^) is mainly produced from the on-off path, which reflects the input of rod system and cone system. The a-wave of dark-adapted 3.0 ERG mainly produced from photoreceptor & post-receptoral on pathway, which reflects the function of photoreceptor (McCulloch et al., 2015). One way ANOVO analysis (*F* = 3,406; *P* < 0.0001) revealed that the amplitude of b-wave of dark-adapted 0.01 ERG decreased dramatically 1 day after Tail vein injection of MMS and the amplitude is the lowest at 7 days compared with normal retina, *P* < 0.0001 (Figure 2C). The amplitude of b-wave of dark-adapted 3.0 ERG decreased dramatically at 1 day after Tail vein injection of MMS and the amplitude is the lowest at 7 days compared with normal retina, *P* < 0.0001 (Figure 2D). Combined with the HE results of retina, we can conclude that the decline of retinal function was faster than the morphology changes of retina.

### The Behavior of Mice Changed After Tail Vein Injection of Methyl Methanesulfonate

**FIGURE 3:**
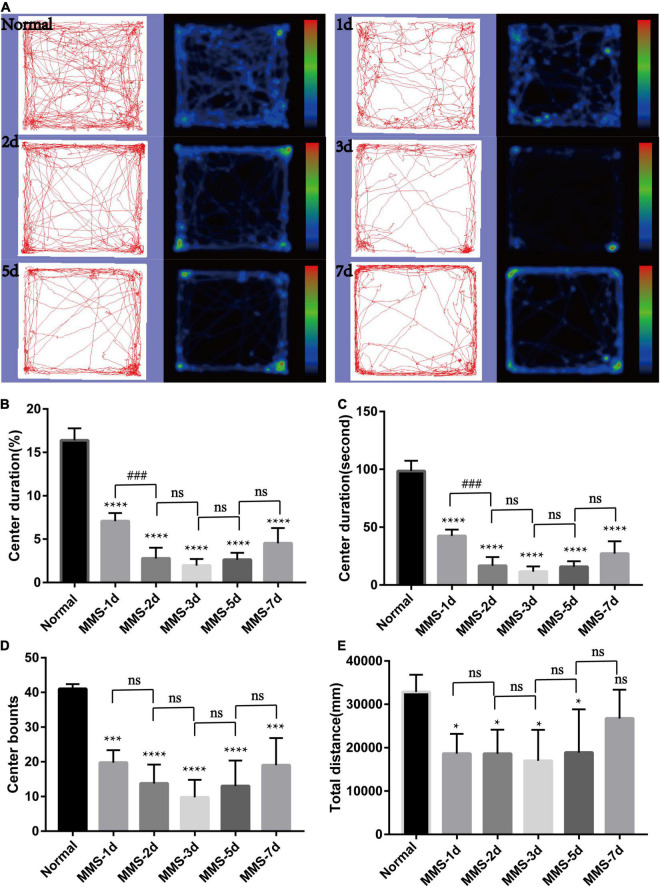
Behavioral changes of mice in open field with time after tail vein injection of MMS. **(A)** Trajectories and density maps of mice without MMS treatment and 1, 2, 3, 5, and 7 days after MMS administration in open field. **(B)** The histogram of the percentage duration of mice in the central area of the open field. One way ANOVA multiple comparisons was analyzed, ****P < 0.0001 for differences compared with controls; ^###^*P* < 0.001 for differences compared with previous time point group. **(C)** The histogram of the duration of mice in the central area of the open field. One way ANOVA multiple comparisons was analyzed, ****P < 0.0001 for differences compared with controls; ^###^*P* < 0.001 for differences compared with previous time point group. **(D)** The histogram of the bounts of mice in the central area of the open field. One way ANOVA multiple comparisons was analyzed, ****P < 0.0001 for differences compared with control, ***P < 0.001 for differences compared with controls. **(E)** The histogram of the total distance of mice in the open field. One way ANOVA multiple comparisons was analyzed, *P < 0.05 for differences compared with controls, *n* = 6.

We speculated that MMS-induced retinal photoreceptor degeneration would seriously affect the visual acuity of mice and then their behavior. Therefore, we used open field test to detect the behavioral changes of mice at day 1, day 2, day 3, day 5, and day 7 after MMS injection (Figure 3). After MMS injection, the total distance of mice in the open field, the number of times (bounts) to the central area and the duration in the central area decreased compared with normal mice (Figures 3B–D), which showed that the changes of visual acuity in mice significantly affected their behavior. One way ANOVA multiple comparisons (*F* = 129.8, *P* < 0.0001) was to analyse the percentage duration of mice stay in the central area of the open field, *P* < 0.0001 for differences compared with controls; *P* < 0.001 for differences compared with previous time point group. One way ANOVA multiple comparisons (*F* = 80.77, *P* < 0.0001) was to analyse the duration of mice stay in the central area of the open field, *P* < 0.0001 for differences compared with Normal; *P* < 0.001 for differences compared with previous time point group. One way ANOVA multiple comparisons (*F* = 16.32, *P* < 0.0001) was to analyse the bounts of mice to the central area of the open field, *P* < 0.0001 for differences compared with Normal, *P* < 0.001 for differences compared with Normal. One way ANOVA multiple comparisons (*F* = 3.632, *P* < 0.05) was to analyse the total distance of mice in the open field, *P* < 0.05 for differences compared with Normal. It can be seen from the four indicators that the behavior of mice is the worst 3 days after MMS injection, and then there is a recovery, which suggests that the vision of mice may have a slight adaptation period.

### Retinal Photoreceptor Cell Death Is Accompanied by Apoptosis After Tail Vein Injection of Methyl Methanesulfonate

**FIGURE 4:**
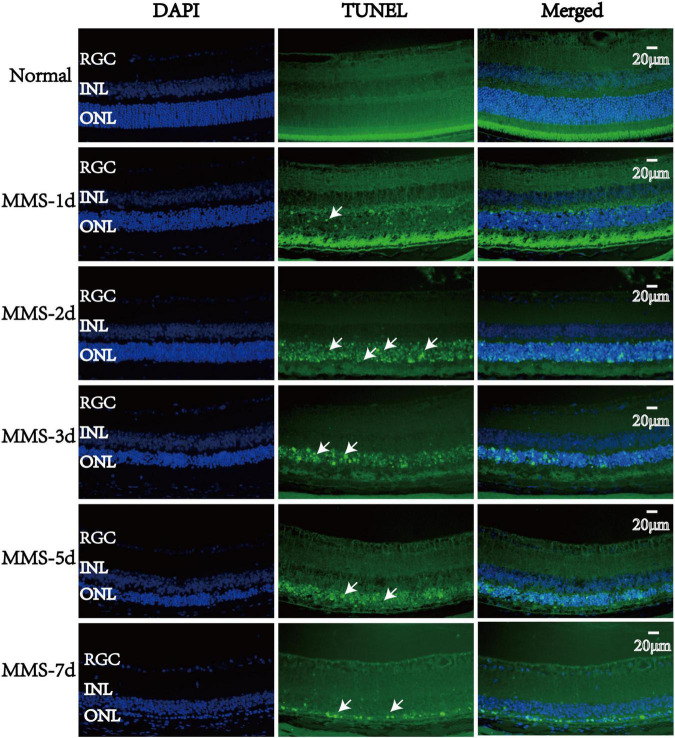
The apoptosis changes of retinal photoreceptors with time after tail vein injection of MMS. The nucleus was labeled with blue fluorescence (DAPI), and the reactive apoptotic cells were labeled with green fluorescence (TUNEL). White arrows indicated apoptotic cells.

TUNEL staining was used to measure the apoptotic process of retinal photoreceptors at day 1, day 2, day 3, day 5, and day 7 after MMS injection (Figure 4). We found that the apoptosis of retinal photoreceptor cells increased from the second day after MMS injection and reached the peak on the third day, while apoptotic cells had been basically eliminated on the seventh day (Figure 4).

### Mechanistic Target of Rapamycin Was Activated in Methyl Methanesulfonate-Induced Retinal Photoreceptor Degeneration

**FIGURE 5:**
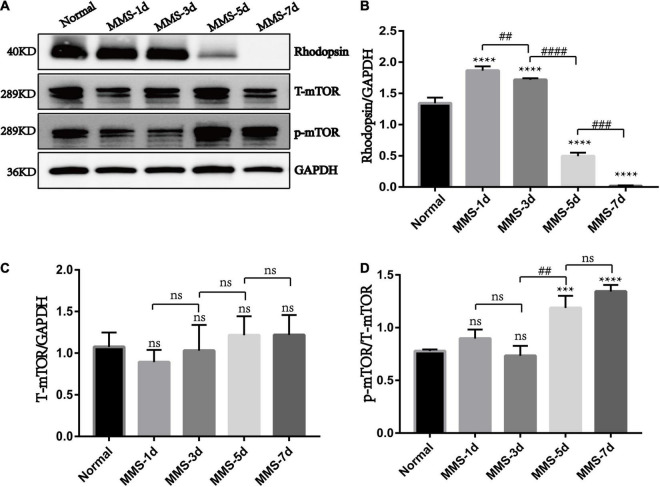
The expression of rhodopsin, mTOR and p-mTOR after tail vein injection of MMS. **(A)** Representative WB images of rhodopsin, Total mTOR (T-mTOR) and p-mTOR. **(B)** The histogram of the immune blotting gray value of rhodopsin/GAPDH. One way ANOVA was analyzed, ****P < 0.0001 for difference between Normal group and MMS injection group, ^##^*P* < 0.01 for difference between MMS-1d and MMS-3d, ^####^*P* < 0.0001 for difference between MMS-3d and MMS-5d, ^###^*P* < 0.001 for difference between MMS-5d and MMS-7d, *n* = 3. **(C)** The histogram of the immune blotting gray value of T-mTOR/GAPDH. One way ANOVA was analyzed, *n* = 3. **(D)** The histogram of the immune blotting gray value of p-mTOR/T-mTOR. One way ANOVA was analyzed, ***P < 0.001 for difference between Normal and MMS-5d, ****P < 0.0001 for difference between Normal and MMS-7d, ^##^*P* < 0.01 for difference between MMS-3d and MMS-5d, *n* = 3.

Our results showed that the expression of rhodopsin which is the marker of retinal rod cells decreased gradually with the injection of MMS and was almost undetectable at 7 days (*P* < 0.0001), which displayed serious loss of rod cells (Figures 5A,B). We also detected that the expression of phosphorylated mTOR (p-mTOR) increased during retinal photoreceptor injury, which was obvious at 5 days (*P* < 0.001) and 7 days (*P* < 0.0001) after MMS injection (Figures 5A,C,D). This suggests that p-mTOR is activated during photoreceptor injury.

### Protective Effects of Rapamycin on Retina at 5 Days After Tail Vein Injection of Methyl Methanesulfonate

**FIGURE 6:**
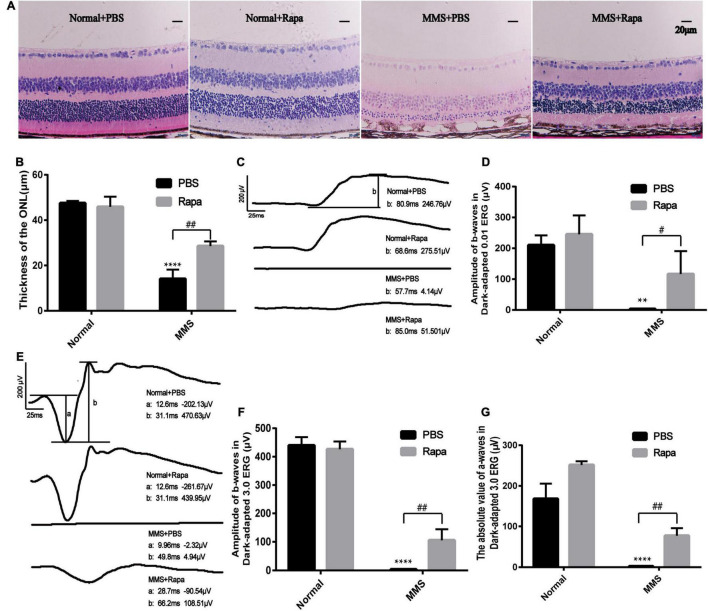
Protective effects of rapamycin on retina at 5 days after tail vein injection of MMS. **(A)** HE staining of the retina in four groups: Normal + PBS, Normal + Rapa, MMS + PBS, MMS + Rapa. **(B)** The histogram of the thickness of the ONL. Two way ANOVA was analyzed, **P < 0.01 for difference between the MMS + PBS and the Normal + PBS, ^#^*P* < 0.05 for difference between the MMS + Rapa and the MMS + PBS, *n* = 3/group. **(C)** The representative b-wave of dark-adapted 0.01 ERG of the mice in four groups: Normal + PBS, Normal + Rapa, MMS + PBS, and MMS + Rapa. **(D)** The histogram of the b-wave of dark-adapted 0.01 ERG. Two way ANOVA was analyzed, ****P < 0.0001 for difference between the MMS + PBS and the Normal + PBS, ^##^*P* < 0.01 for difference between the MMS + Rapa and the MMS + PBS, *n* = 3/group. **(E)** The representative b-wave of dark-adapted 3.0 ERG of the mice in four groups: Normal + PBS, Normal + Rapa, MMS + PBS, and MMS + Rapa. **(F)** The histogram of the b-wave of dark-adapted 3.0 ERG. Two way ANOVA was analyzed, ****P < 0.0001 for difference between the MMS + PBS and the Normal + PBS, ^##^*P* < 0.01 for difference between the MMS + Rapa and the MMS + PBS, *n* = 3/group. **(G)** The histogram of the absolute value of a-wave of dark-adapted 3.0 ERG. Two way ANOVA was analyzed, ****P < 0.0001 for difference between the MMS + PBS and the Normal + PBS, ^##^*P* < 0.01 for difference between the MMS + Rapa and the MMS + PBS, *n* = 3/group.

Because the expression of p-mTOR increased during MMS induced photoreceptor injury, we decided to use rapamycin, an inhibitor of mTOR to see whether it could reverse this injury. The experimental mice were divided into four groups with three mice in each group. The Normal + PBS group was only injected with PBS. The Normal + Rapa group was only injected with rapamycin. The MMS + PBS group was only injected with MMS. The MMS + Rapa group was injected with rapamycin intraperitoneally every day from the day before MMS injection until 5 days after MMS injection. Figure 6A showed the representative retinal morphology observed by H&E staining. Two-way ANOVA analysis revealed that both MMS (*F* = 185.2, *P* < 0.0001) and rapamycin (*F* = 11.78, *P* < 0.01) had effect on the thickness of the ONL, and there was a significant interaction between them (*F* = 18.72, *P* < 0.01). The thickness of ONL decreased in MMS + PBS group compared with the Normal + PBS group (*P* < 0.0001), while the thickness of ONL in MMS + Rapa group was significantly thicker than that in the MMS + PBS group (*P* < 0.01; Figures 6A,B), which exhibited the protective effect of rapamycin on the morphology of retinal photoreceptor. There was no significant difference in the thickness of ONL between the Normal + Rapa mice and the Normal + PBS mice (*P* > 0.05), indicating that rapamycin itself does not damage the retinal photoreceptor cells of mice.

There was no significant difference in the b-wave of Dark-adapted 0.01 ERG between the Normal + Rapa mice and the Normal + PBS mice (*P* > 0.05), indicating that rapamycin itself had no negative effect on retinal function of mice (Figures 6C,D). While the b-wave in Dark-adapted 0.01 ERG in the MMS + Rapa group was significantly higher than that in the MMS + PBS group (*P* < 0.05; Figures 6C,D), which displayed the protective effect of rapamycin on the function of mouse retinal rod cells. There was no significant difference in the amplitude of b-wave in Dark-adapted 3.0 ERG between the Normal + Rapa mice and the Normal + PBS mice (*P* > 0.05), indicating that rapamycin itself had no negative effect on retinal function of mice (Figures 6E,F). While the amplitude of b-wave in dark-adapted 3.0 ERG in the MMS + Rapa group was significantly higher than that in the MMS + PBS group (*P* < 0.01; Figures 6E,F), which displayed the protective effect of rapamycin on the function of mouse retinal photoreceptor. The absolute value of a-wave in dark-adapted 3.0 ERG between in the Normal + Rapa mice was bigger than that in the Normal + PBS mice (*P* < 0.01), indicating that rapamycin itself had positive effect on retinal photoreceptor function of mice (Figures 6E,G). Also, the absolute value of a-wave in dark-adapted 3.0 ERG in the MMS + Rapa group was significantly bigger than that in the MMS + PBS group (*P* < 0.01; Figures 6E,F), which displayed the protective effect of rapamycin on the function of mouse retinal photoreceptor. We also found that rapamycin could improve the retinal morphology and retinal function at 7 days after MMS treatment (Supplementary Figure 1).

**FIGURE 7:**
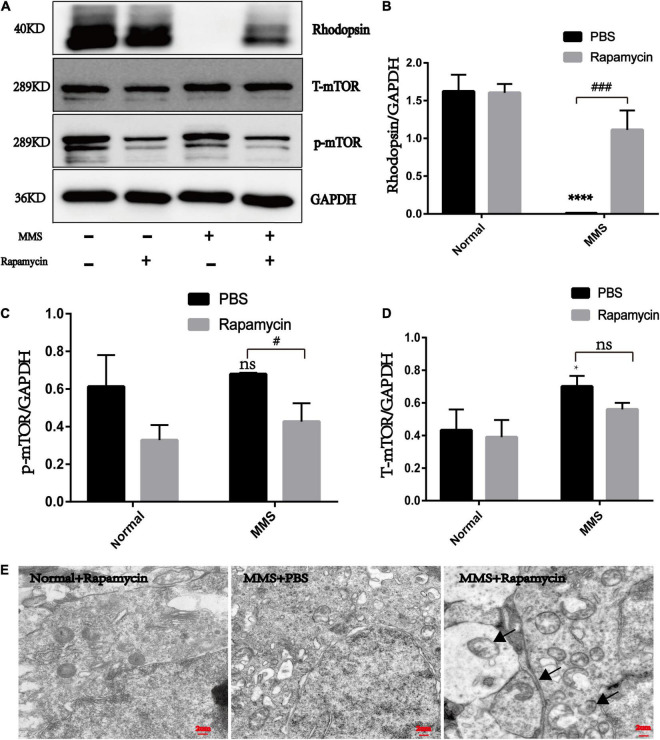
The effect of rapamycin on the expression of rhodopsin, mTOR and p-mTOR. **(A)** Representative WB images of rhodopsin, mTOR and p-mTOR. **(B)** The histogram of the immune blotting gray value of rhodopsin/GAPDH. One way ANOVA was analyzed, ****P < 0.0001 for the difference between the MMS + PBS and the Normal + PBS, ^###^*P* < 0.001 for the difference between the MMS + PBS and the MMS + Rapa, *n* = 3. **(C)** The histogram of the immune blotting gray value of p-mTOR/GAPDH. One way ANOVA was analyzed, ^#^*P* < 0.05 for the difference between the MMS + PBS and the MMS + Rapa, *n* = 3. **(D)** The histogram of the immune blotting gray value of T-mTOR/GAPDH. One way ANOVA was analyzed, *P < 0.05 for the difference between the MMS + PBS and the Normal + PBS, *n* = 3. **(E)** Representative TEM images of retinal photoreceptor in the Normal + Rapa, the MMS + PBS and the MMS + Rapa. Black arrows represent autophagosome.

The expression of rhodopsin in the MMS + Rapa group increased compared with the MMS + PBS group (*P* < 0.001), which was the molecular evidence for the improvement of retinal function and morphology (Figures 7A,B). The expression of p-mTOR in the MMS + Rapa group decreased compared to the MMS + PBS group (*P* < 0.05) while the expression of T-mTOR didn’t have significance difference (Figure 7D), which showed that the rapamycin inhibited the expression of p-mTOR (Figures 7A,C). We also detected autophagosome in the MMS + Rapa group by transmission electron microscope (TEM) (Figure 7E), which suggests that rapamycin may delay the death progress of retinal photoreceptor cells by inhibiting mTOR and then activating autophagy to remove the waste in the process of retinal photoreceptor cell death.

### Rapamycin Improved the Behavior of Mice at 5 Days After Tail Vein Injection of Methyl Methanesulfonate

**FIGURE 8:**
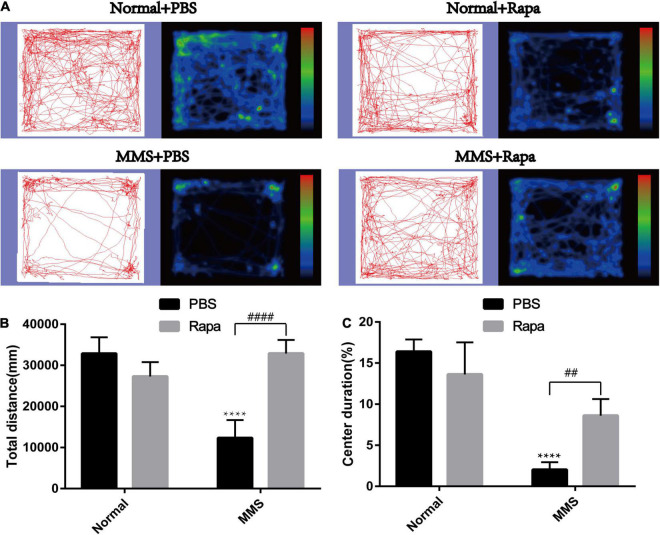
Effect of rapamycin on the behavioral changes of mice in open field at 5 days after tail vein injection of MMS. **(A)** Trajectories and density maps of mice in four groups: Normal + PBS, Normal + Rapa, MMS + PBS, MMS + Rapa. **(B)** The histogram of the total distance of the mice. Two way ANOVA was analyzed, ****P < 0.0001 for difference between MMS + PBS and Normal + PBS, ^####^*P* < 0.0001 for difference between MMS + Rapa and MMS + PBS, *n* = 6. **(C)** The histogram of the percentage of center duration of mice. Two way ANOVA was analyzed, ****P < 0.0001 for difference between MMS + PBS and Normal + PBS, ^##^*P* < 0.01 for difference between MMS + Rapa and MMS + PBS, *n* = 6/group.

**FIGURE 9:**
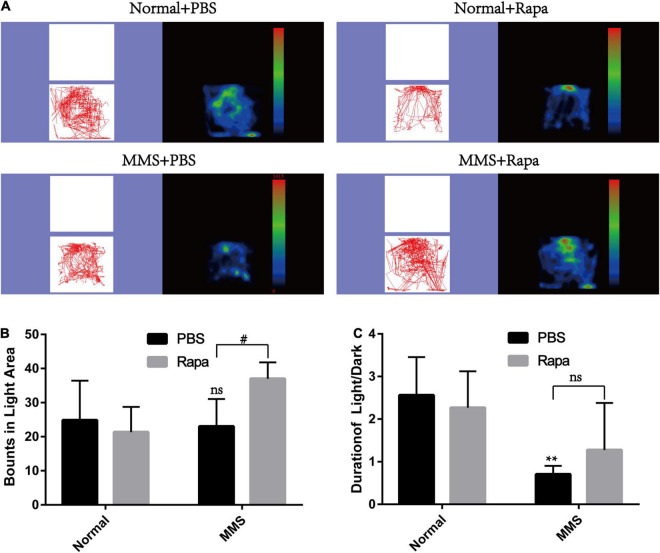
Effect of rapamycin on the behavioral changes of mice in Light/Dark transition at 5 days after tail vein injection of MMS. **(A)** Trajectories and density maps of mice in four groups: Normal + PBS, Normal + Rapa, MMS + PBS, MMS + Rapa. **(B)** The histogram of the number of times to light area of the mice. Two way ANOVA was analyzed, ^#^*P* < 0.05 for difference between MMS + Rapa and MMS + PBS, *n* = 6. **(C)** The histogram of the percentage of center duration of mice. Two way ANOVA was analyzed, **P < 0.01 for difference between MMS + Rapa and MMS + PBS, *n* = 6.

The experimental grouping was consistent with the above. There was no significant difference in the total distance and central duration between the Normal + Rapa mice and the Normal + PBS mice in the open field test (*P* > 0.05), indicating that rapamycin itself had no negative effect on the behavior the mice (Figure 8). While the total distance (*P* < 0.0001) and central duration (*P* < 0.01) of mice in the MMS + Rapa group was significantly longer than that in the MMS + PBS group (Figure 8), which displayed the positive effect of rapamycin on the behavior of the mice. There was no significant difference in the number of times to light area and the ratio of time in the dark area to time in the light area between the Normal + Rapa group and the Normal + PBS group in the open field test (*P* > 0.05), indicating that rapamycin itself had no negative effect on the behavior of the mice (Figure 9). The number of times to light area of mice in the MMS + Rapa group was significantly more than that in the MMS + PBS group (*P* < 0.05), while the ratio of time in the dark area to time in the light between these two groups showed no differences (Figure 9).

## Discussion

Retinal degenerative diseases which are characterized by progressive degeneration of retinal neurons seriously affect human vision. Retinal degenerative diseases mainly include RP dominated by photoreceptor cell degeneration, age-related macular degeneration (AMD) dominated by RPE cell degeneration and glaucoma dominated by ganglion cell degeneration and other diseases (Madeira et al., 2015). At present, there are different kinds of animal models for different retinal degenerative diseases (Collin et al., 2020). The animal models of RP include gene mutant mice such as rd1, rd10 and cpfl1 (Gagliardi et al., 2019), pigs and mice with retinal photoreceptor degeneration caused by MNU (Chen et al., 2014; Choi et al., 2021) and retinal photoreceptor degeneration mice caused by light (Grimm and Reme, 2019). Previous studies have found that nitrosourea alkylating agent MNU and alkylsulfonate alkylating agent MMS can specifically cause the degeneration of mouse retinal photoreceptor cells without affecting other retinal cells (Meira et al., 2009). The process of MNU induced degeneration of mouse retinal photoreceptors has been revealed (Tao et al., 2015), but the time axis changes of mouse retinal photoreceptors induced by MMS are still unknown.

Here, we explored the impact of single tail vein injection with MMS (60 mg/kg) on the retina of adult male mice. Firstly, we obtained the eyeball of mice at different time to see the morphology changes of retina after MMS injection. There were no difference of the thickness of retinal ganglion cell layer and the inner nuclear layer between MMS group and Normal, while the ONL and outer segment/inner segment layer where retinal photoreceptor lied decreased as time and became thinnest at 10 days. The function of photoreceptor decreased rapidly at 1 day after MMS injection, which showed that the functional injury precedes morphological injury of MMS on retina. TUNEL images showed that the apoptosis of mouse photoreceptor cells was the most obvious at 3 days after MMS injection. The behavior of mice in MMS group appeared abnormal compared with the normal mice at 1 day, which indicated that the impairment of retina influenced the mice behavior. Although the MMS (60 mg/kg) have no significant influence in the morphology of mice brain, we can’t exclude the possibility that MMS impaired the function of brain, which also could influence the mice behavior.

Mechanistic target of rapamycin is located in the central pathway of growth and energy metabolism and changes in a variety of diseases in response to pathological changes (Zoncu et al., 2011). The nutrient-sensing kinase mTOR is an important regulator of nutrient regulation and autophagy (Alers et al., 2012). A nutrient-sensitive complex in mTORC1 mainly contains mTOR and regulatory-associated protein of TOR (raptor) which are sensitive to nutrition, energy and metabolism and affect the kinase activity of mTOR and its downstream effector (Kim et al., 2002). The main substrates catalyzed by mTORC1 are S6 Kinase 1 (S6K1) and eIF-4E binding protein 1 (4E-BP1). Both of which are mainly responsible for the translation process of mRNA, promote protein synthesis and stimulate autophagy regulators FIP200 and Atg101 (Brown et al., 1995; Hara et al., 1997, 2008; Hosokawa et al., 2009). mTORC2, which is mainly comprised mTOR, rapamycin-insensitive companion of mTOR (rictor) and GβL, regulates the actin cytoskeleton and mainly activates the Ser/Thr protein kinases of protein kinase C-alpha (PKC-α), which share the hydrophobic motif phosphorylation site with S6K (Sarbassov et al., 2004).

Our results showed that mTOR was activated during retinal degeneration induced by MMS, which indicated that the mTOR participated this degeneration process, which may be involved in metabolism or autophagy. As an immunosuppressant, rapamycin can bind to intracellular receptor FKBP12, interfere with substrates recruitment and active sites, and then inhibit mTOR kinase activity (Choi et al., 1996). By using the inhibitor of mTOR the thickness of ONL where retinal photoreceptor was located become thicker, the function of retinal photoreceptor become better and the expression of the biomarker protein of retinal photoreceptor increased, which exhibited that rapamycin improved the retinal morphology and function impaired by MMS. In addition, the behavior of mice become more active in the open field and tried more times to explored into the light area in the Dark/Light transition field, which reflected that the rapamycin affect the mice behavior. The chaperone protein of mTORC1 is raptor and the chaperone protein of mTORC 2 is rictor, which determines that mTORC1 is sensitive to rapamycin and mTORC 2 is not (Wullschleger et al., 2006). Therefore, the rapamycin may inhibited the mTORC1 to protect retinal photoreceptor.

Since one of the main functions of mTORC1 is to inhibit autophagy, inhibition of mTORC1 by small molecules or drugs can activate autophagy (Yang et al., 2013). Proper and accurate autophagy can remove discarded organelles and replenish the energy of cells (Feng et al., 2019). It was discovered that the activation of autophagy protected retinal ganglion cells in the acute glaucoma model caused by optic nerve extrusion or transection (Kim et al., 2008; Rodriguez-Muela et al., 2012). In a diabetic retinopathy model, autophagosome formation of Müller cells increased, lysosomal hydrolase activity decreased, p62/SQTSM1 substrate accumulated and cell apoptosis increased. However, inhibition of mTOR activated autophagy with rapamycin improved the above conditions and reduced cell apoptosis (Lopes de Faria et al., 2016). In the AMD model, rapamycin inhibits mTORC1 to activate autophagy which eliminates lipofuscin deposition due to the phagocytosis of photoreceptor outer segments by pigment epithelial cells, which delays the progression of AMD (Mitter et al., 2014). Therefore, we guessed that is autophagy involved in the retinal protection process of rapamycin? We used TEM to detect the autophagy after rapamycin treatment and found the autophagosome in rapamycin + MMS group, which indicated that rapamycin may activate autophagy by inhibiting mTORC1, and the activated autophagy may delay the progress of photoreceptor cell death by removing some harmful substances in the process of photoreceptor cell damage. This suggests that mTORC1 may be a target for the treatment of RP and has guiding significance for clinical practice.

In other RP models, targeting mTOR has also achieved certain results in the treatment of RP. In the P23H mutant rod opsin mice, rapamycin could reduce the rod opsin aggregates generated from P23H mutant by increasing autophagy (Mendes and Cheetham, 2008). In the P23H-3 RHO rats, rapamycin was used to inhibit the expression of mTOR and exhibited neuronal protection for retinal photoreceptor although the expression of autophagy protein didn’t have any changes (Sizova et al., 2014). Inhibition of tuberin which is one inhibitor of mTOR pathway preserved the retinal photoreceptor number and function in a preclinical RP model mutant Pde6b^H620Q^/Pde6b^H620Q^ (Tsang et al., 2014). The m-TOR pathway was activated in Pde6b^rd1^/Pde6b^rd1^ mice and treatment with rapamycin significantly protect the morphology of retinal photoreceptor, which may through the activation of autophagy (Yang et al., 2021). Taken together, further efforts are needed to comprehensively and deeply understand the protective mechanism of targeting mTOR on retina to advance clinical trials for RP.

## Data Availability Statement

The original contributions presented in the study are included in the article/Supplementary Material, further inquiries can be directed to the corresponding author.

## Ethics Statement

The animal study was reviewed and approved by Animal Research Ethics Committee of Zhengzhou University.

## Author Contributions

MZ performed most of the experiments, analyzed the data, wrote the original draft, and edited the manuscript. HL performed model building and functional evaluation. NY performed part of the behavioral experiments. G-HP revised the manuscript, provided support, and supervised the project. All authors have read and agreed to the published version of the manuscript.

## Conflict of Interest

The authors declare that the research was conducted in the absence of any commercial or financial relationships that could be construed as a potential conflict of interest.

## Publisher’s Note

All claims expressed in this article are solely those of the authors and do not necessarily represent those of their affiliated organizations, or those of the publisher, the editors and the reviewers. Any product that may be evaluated in this article, or claim that may be made by its manufacturer, is not guaranteed or endorsed by the publisher.

## Supplementary Material

Supplementary Figure 1 Protective effects of rapamycin on retina at 7 days after tail vein injection of MMS. **(A)** HE staining of the retina in four groups: Normal + PBS, Normal + Rapa, MMS + PBS, and MMS + Rapa. **(B)** The histogram of the thickness of the ONL. Two way ANOVA was analyzed, ^∗∗∗∗^*P* < 0.0001 for difference between the MMS + PBS and the Normal + PBS, ^###^*P* < 0.001 for difference between the MMS + Rapa and the MMS + PBS, *n* = 3/group. **(C)** The representative b-wave of dark-adapted 3.0 ERG of the mice in four groups: Normal + PBS, Normal + Rapa, MMS + PBS, and MMS + Rapa. **(D)** The histogram of the b-wave of dark-adapted 3.0 ERG. Two way ANOVA was analyzed, ^∗∗∗∗^*P* < 0.0001 for difference between the MMS + PBS and the Normal + PBS, ^#^*P* < 0.05 for difference between the MMS + Rapa and the MMS + PBS, *n* = 3/group.
