# Supplementary figures and images for: Rapamycin Improved Retinal Function and Morphology in a Mouse Model of Retinal Degeneration
Source: Front Neurosci. 2022 Feb 28;16:846584. doi: 10.3389/fnins.2022.846584 (PMC8919089; doi:10.3389/fnins.2022.846584)

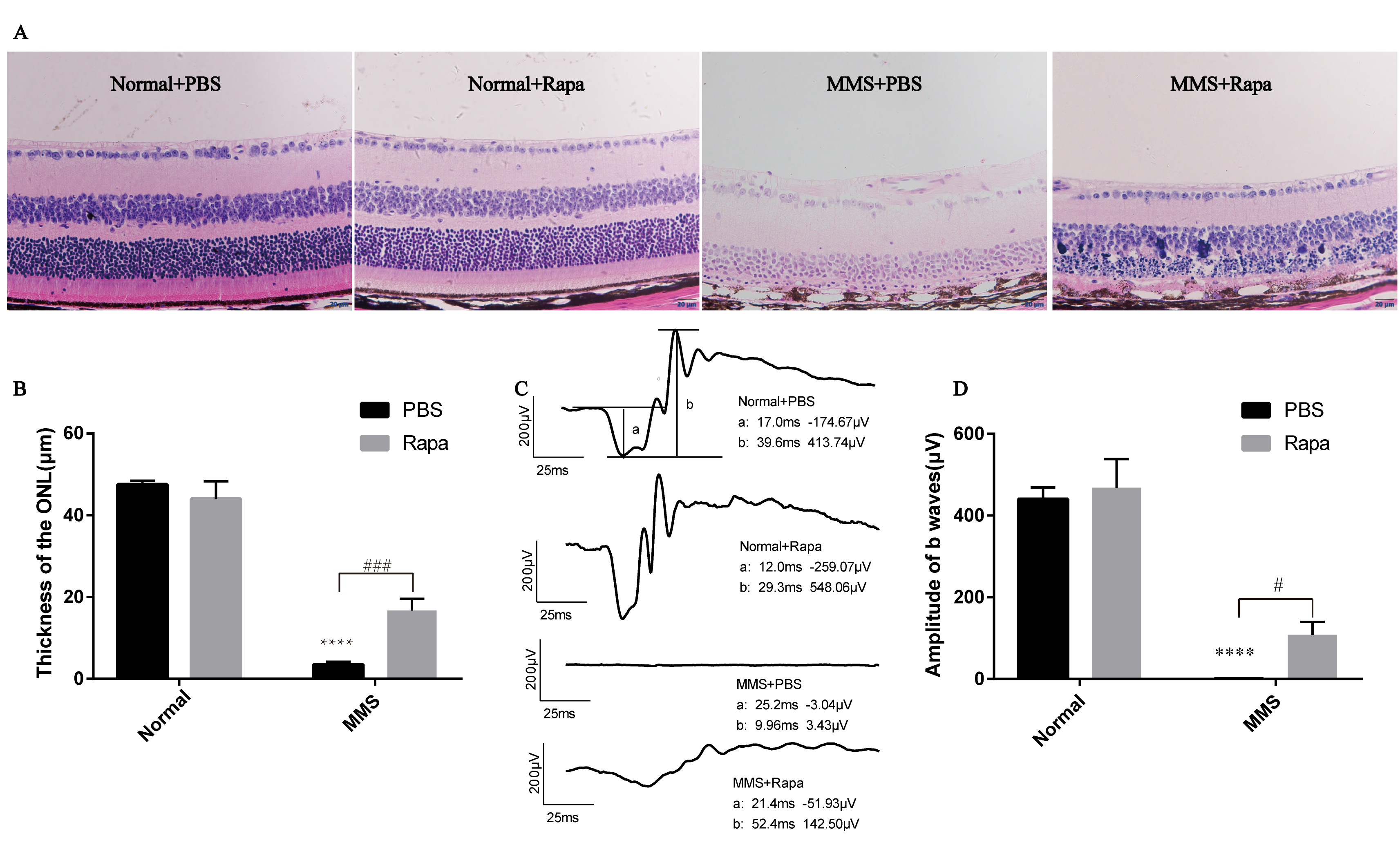

Supplement: Supplementary Figure 1 — Protective effects of rapamycin on retina at 7 days after tail vein injection of MMS. (A) HE staining of the retina in four groups: Normal + PBS, Normal + Rapa, MMS + PBS, and MMS + Rapa. (B) The histogram of the thickness of the ONL. Two way ANOVA was analyzed, ∗∗∗∗P < 0.0001 for difference between the MMS + PBS and the Normal + PBS, ###P < 0.001 for difference between the MMS + Rapa and the MMS + PBS, n = 3/group. (C) The representative b-wave of dark-adapted 3.0 ERG of the mice in four groups: Normal + PBS, Normal + Rapa, MMS + PBS, and MMS + Rapa. (D) The histogram of the b-wave of dark-adapted 3.0 ERG. Two way ANOVA was analyzed, ∗∗∗∗P < 0.0001 for difference between the MMS + PBS and the Normal + PBS, #P < 0.05 for difference between the MMS + Rapa and the MMS + PBS, n = 3/group. [file Image_1.TIF]
